# Author Correction: Loss of BCL9/9l suppresses Wnt driven tumourigenesis in models that recapitulate human cancer

**DOI:** 10.1038/s41467-019-09465-7

**Published:** 2019-03-26

**Authors:** David M. Gay, Rachel A. Ridgway, Miryam Müller, Michael C. Hodder, Ann Hedley, William Clark, Joshua D. Leach, Rene Jackstadt, Colin Nixon, David J. Huels, Andrew D. Campbell, Thomas G. Bird, Owen J. Sansom

**Affiliations:** 1 Cancer Research UK Beatson Institute, Garscube Estate, Switchback Road, Glasgow, G61 1BD UK; 2 Institute of Cancer Sciences, University of Glasgow, Garscube Estate, Glasgow, G61 1QH UK; 3 MRC Centre for Inflammation Research, The Queen’s Medical Research Institute, University of Edinburgh, Edinburgh, EH16 4TJ UK; 4 Present Address: Academic Medical Center (AMC), University of Amsterdam, Amsterdam, 1105 AZ The Netherlands

Correction to: *Nature Communications* 10.1038/s41467-019-08586-3, published online 13 February 2019

The original version of this Article contained an error in the spelling of the author Miryam Müller, which was incorrectly given as Miryam Müeller. This has now been corrected in both the PDF and HTML versions of the Article.

