# Supplementary material for: Loss of BCL9/9l suppresses Wnt driven tumourigenesis in models that recapitulate human cancer
Source: Nat Commun. 2019 Feb 13;10:723. doi: 10.1038/s41467-019-08586-3 (PMC6374445; doi:10.1038/s41467-019-08586-3)
Supplement: Supplementary file 1 — Supplementary Information [file 41467_2019_8586_MOESM1_ESM.pdf]

Supplementary information

Loss of BCL9/9l suppresses Wnt driven tumourigenesis in models that recapitulate human cancer

Gay et al.,

Supplementary figure 1

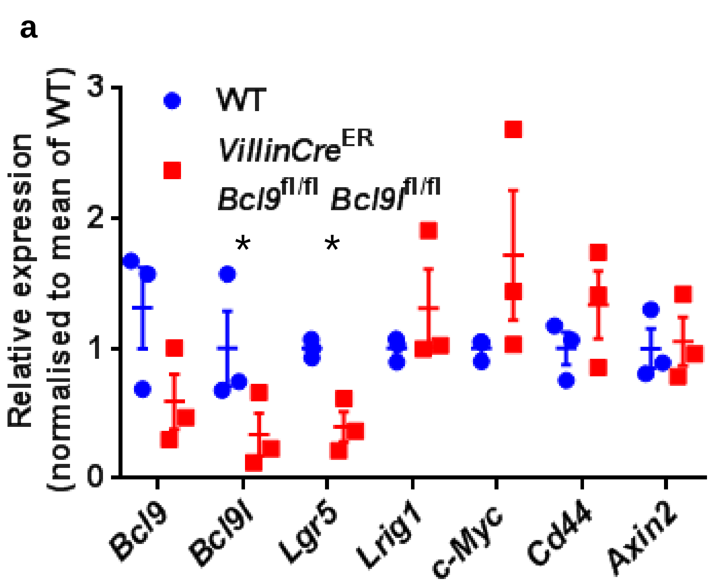

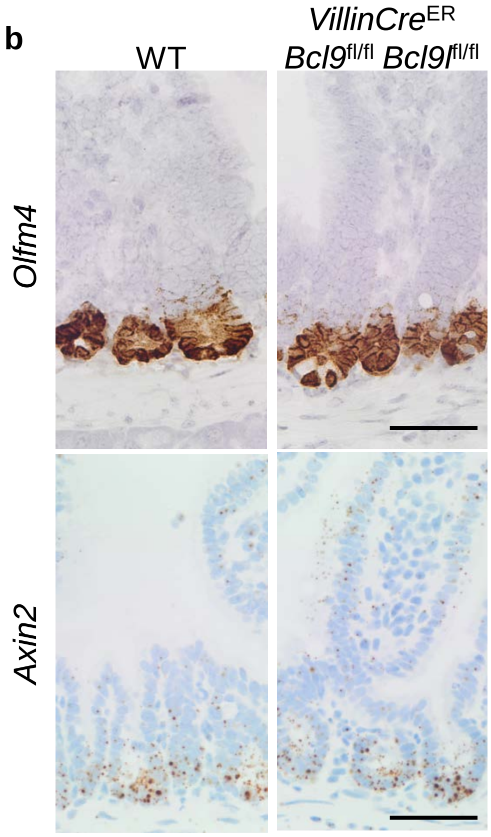

Supplementary figure 1: *Bcl9/9l* deletion does not affect other stem cell markers

a. qPCR for Wnt target genes and intestinal stem cell markers from small intestinal tissue of WT and *VillinCre*^{ER} *Bcl9*$^{fl/fl}$ *Bcl9l*$^{fl/fl}$ mice, sampled four days post tamoxifen injection. Data displayed as relative to the mean of the WT group. n=3 per group, one-way Mann-Whitney *U* test, *P*=0.04 (*). Data displayed as mean + SEM.

b. Representative *Olfm4* and *Axin2* RNAscope staining of small intestinal sections from mice described in a. Scale bar = 50µm.

Supplementary figure 2

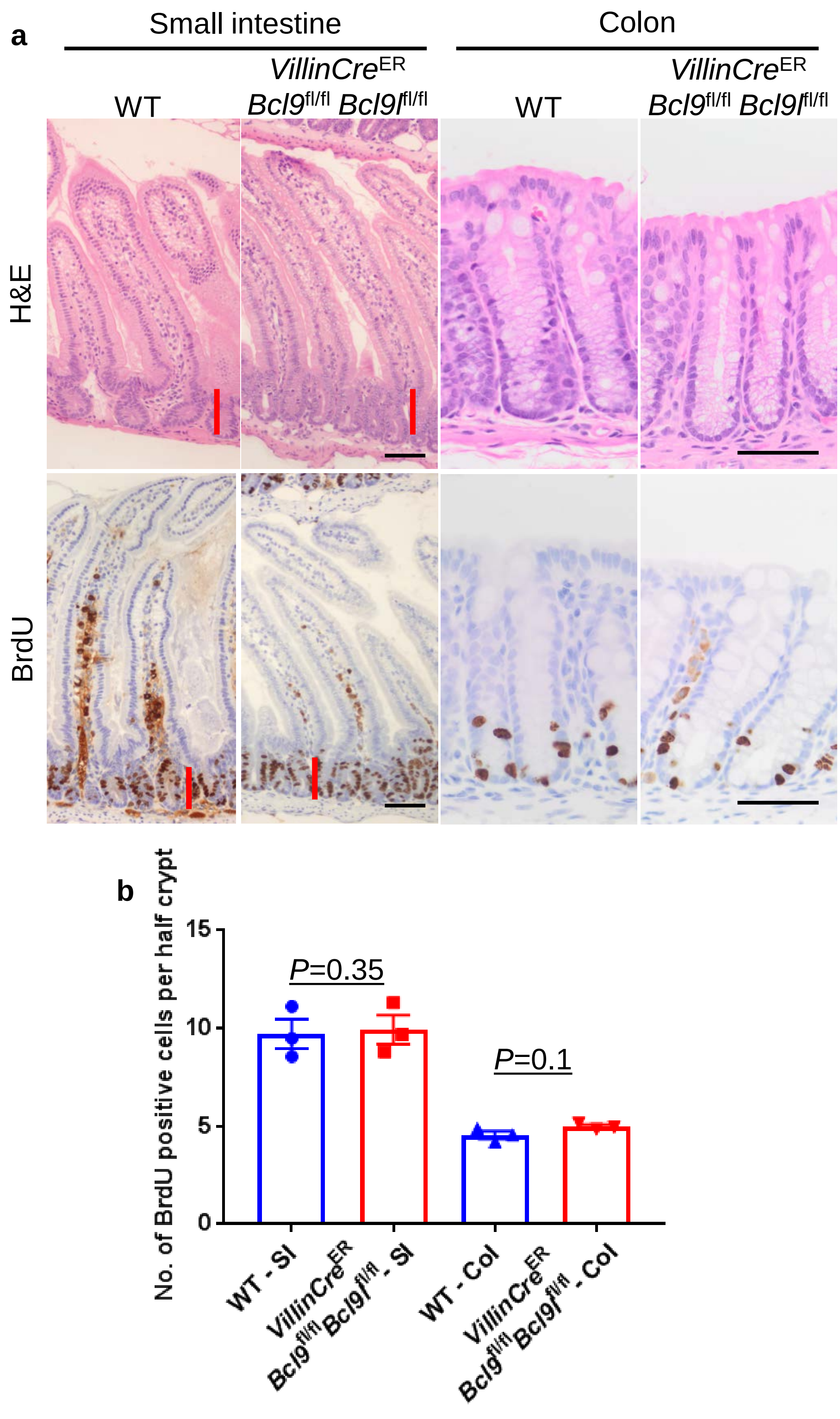

Supplementary figure 2: BCL9/9l are dispensable for normal intestinal homeostasis

a. Representative H&E and BrdU staining of small intestines (left panel) and colons (right panel) from Cre-induced Wildtype (WT) and *VillinCre*^{ER} *Bcl9*$^{fl/fl}$ *Bcl9l*$^{fl/fl}$ mice sampled four days post Cre-induction. Red bars indicate crypt size. Scale bar = 50 µm.

b. Quantification of proliferation in small intestine and colons of WT and *VillinCre*^{ER} *Bcl9*$^{fl/fl}$ *Bcl9l*$^{fl/fl}$ mice, number of BrdU positive cells per half-crypt was scored, 25 crypts scored per mouse, n=3 per group, one-way Mann-Whitney U test P=0.35 (small intestine) and P=0.1 (colon). Data displayed as mean $\pm$ SEM.

Supplementary figure 3

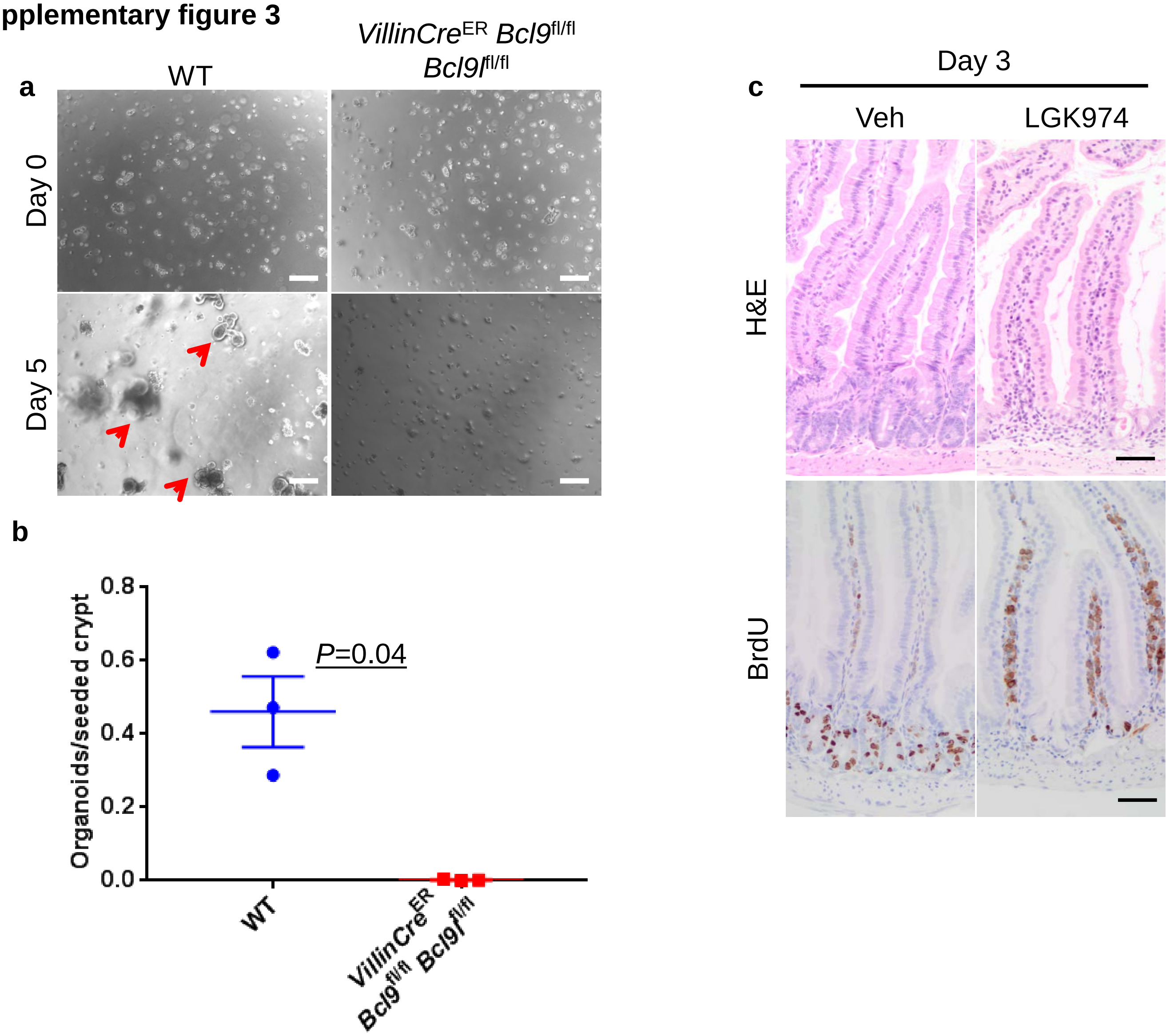

Supplementary figure 3: BCL9/9l are required for intestinal organoid formation

a. Representative images from day 0 and day 5 post-seeding of small intestinal cultures isolated from WT and *VillinCre*^{ER} *Bcl9*$^{fl/fl}$ *Bcl9l*$^{fl/fl}$ mice and seeded four days post Cre-induction. Red arrows indicate budding organoids. Scale bar = 40μm.

b. Quantification of viable small intestinal organoids isolated from mice described in a. 150 crypts seeded per well – total number of viable organoids/seeded crypt scored, n=3 per group one-way Mann-Whitney *U* test, *P*=0.04. Data displayed as mean ± SEM.

c. Representative H&E (upper panel) and BrdU (lower panel) staining of small intestinal sections from Cre-induced *VillinCre*^{ER} *Bcl9*$^{fl/fl}$ *Bcl9l*$^{fl/fl}$ mice treated with 5mg/kg LGK974 or vehicle, twice daily starting 24 hours after tamoxifen injection, sampled at day 3 post-induction. Scale bars = 50μm

Supplementary figure 4

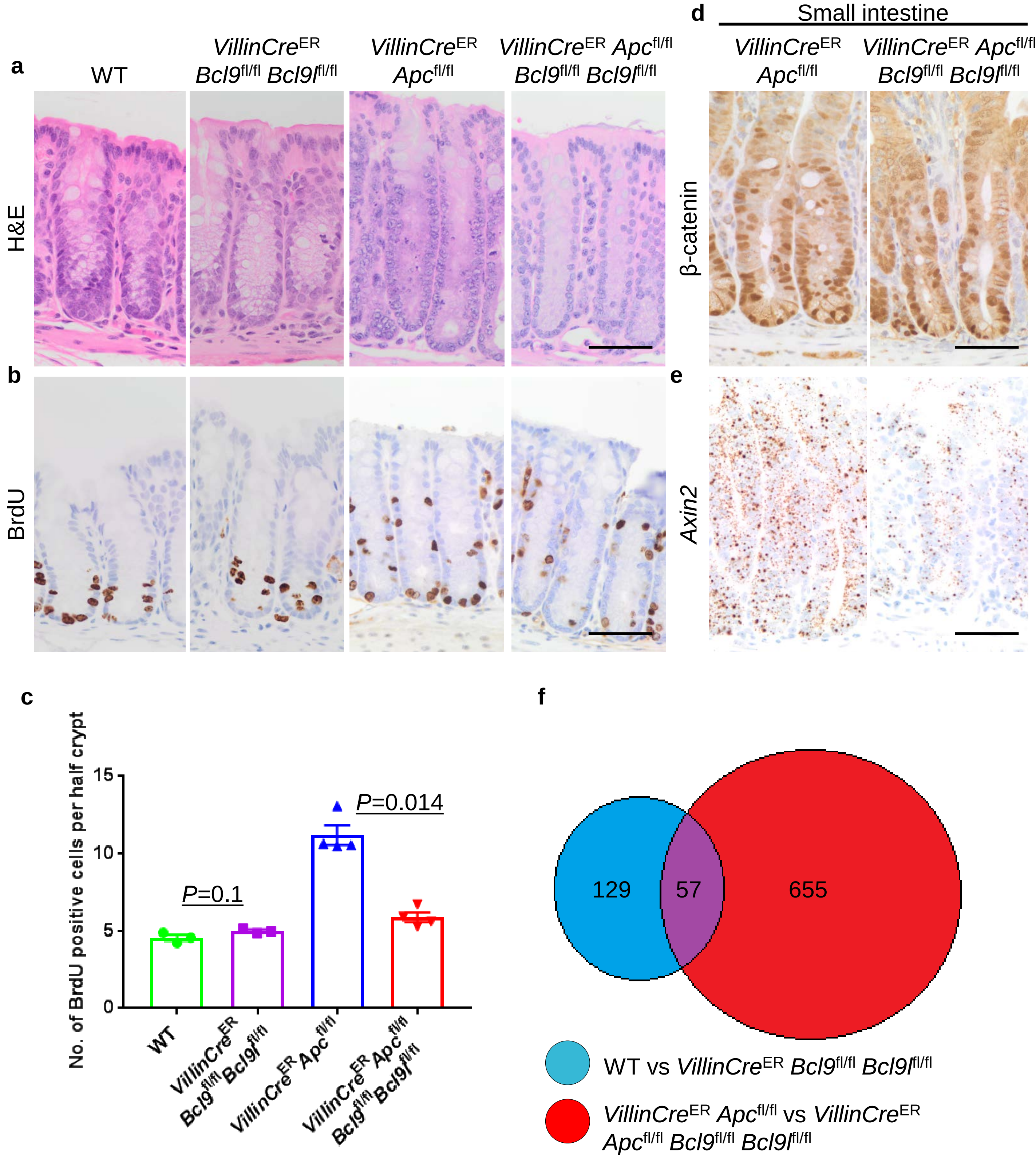

Supplementary figure 4: *Bcl9/9l* deletion suppresses the APC loss crypt progenitor phenotype

a. Representative H&E staining of colons from WT, *VillinCre*^{ER} *Bcl9*$^{fl/fl}$ *Bcl9l*$^{fl/fl}$, *VillinCre*^{ER} *Apc*$^{fl/fl}$ and *VillinCre*^{ER} *Apc*$^{fl/fl}$ *Bcl9*$^{fl/fl}$ *Bcl9l*$^{fl/fl}$ mice sampled four days post Cre-induction. Scale bar = 50µm.

b. Representative BrdU staining of mice described in a. Mice were injected intraperitoneally with BrdU 2 hours prior to being culled. Scale bar = 50µm

c. Quantification of proliferation (BrdU positive cells) in the colons of mice described in a. The number of BrdU-positive cells per half crypt was quantified, 25 crypts per mouse scored, n=3-4 for each group, one-way Mann-Whitney *U* test *P*=0.014 for *VillinCre*^{ER} *Apc*$^{fl/fl}$ vs *VillinCre*^{ER} *Apc*$^{fl/fl}$ *Bcl9*$^{fl/fl}$ *Bcl9l*$^{fl/fl}$ and *P*=0.1 for WT vs *VillinCre*^{ER} *Bcl9*$^{fl/fl}$ *Bcl9l*$^{fl/fl}$. WT data from Supplementary Figure 2a. Data displayed as mean ± SEM.

d. Representative β-catenin staining of small intestines from Cre-induced *VillinCre*^{ER} *Apc*$^{fl/fl}$ and *VillinCre*^{ER} *Apc*$^{fl/fl}$ *Bcl9*$^{fl/fl}$ *Bcl9l*$^{fl/fl}$ mice. Scale bar = 50µm

e. Representative *Axin2*-RNAscope staining of small intestines from mice described in d. Scale bar = 50µm.

f. Venn-diagram comparing overlap of differentially regulated genes from RNAseq on intestinal tissue from WT vs Cre-induced *VillinCre*^{ER} *Bcl9*$^{fl/fl}$ *Bcl9l*$^{fl/fl}$ and *VillinCre*^{ER} *Apc*$^{fl/fl}$ vs *VillinCre*^{ER} *Apc*$^{fl/fl}$ *Bcl9*$^{fl/fl}$ *Bcl9l*$^{fl/fl}$ mice.

Supplementary figure 5

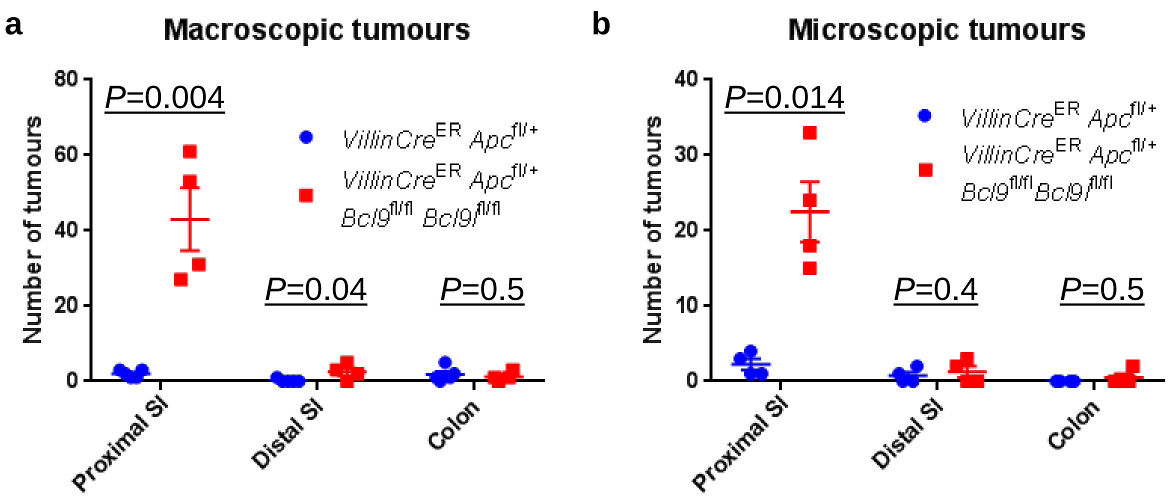

Supplementary figure 5: Deletion of *Bcl9/9l* accelerates proximal small intestinal tumour formation

a. Distribution of macroscopic tumours along the small intestine and colon of Cre-induced *VillinCre*^{ER} *Apc*$^{fl/+}$ and *VillinCre*^{ER} *Apc*$^{fl/+}$ *Bcl9*$^{fl/fl}$ *Bcl9l*$^{fl/fl}$ mice sampled 50 days post-induction, n=5 for *VillinCre*^{ER} *Apc*$^{fl/+}$ and n=4 for *VillinCre*^{ER} *Apc*$^{fl/+}$ *Bcl9*$^{fl/fl}$ *Bcl9l*$^{fl/fl}$, one-way Mann-Whitney *U* test *P*=0.004 (proximal SI), *P*=0.04 (distal SI) and *P*=0.5 (colon). Data displayed as mean ± SEM.

b. Distribution of microscopic tumours along the small intestine and colon of mice described in a, tumours scored from β-catenin stained sections, n=4 per group, one-way Mann-Whitney *U* test *P*=0.014 (proximal SI), *P*=0.4 (distal SI) and *P*=0.5 (colon). Data displayed as mean ± SEM.

Supplementary figure 6

a

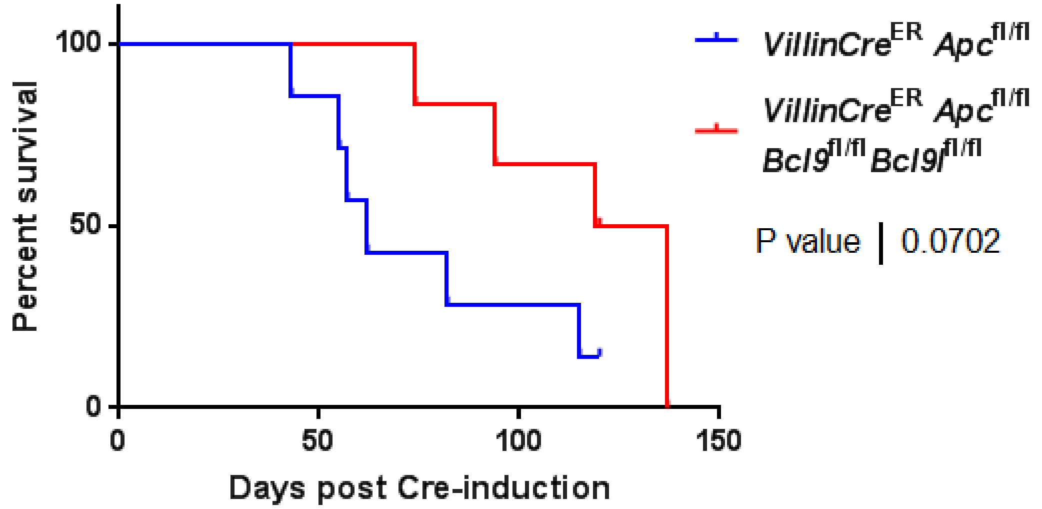

b

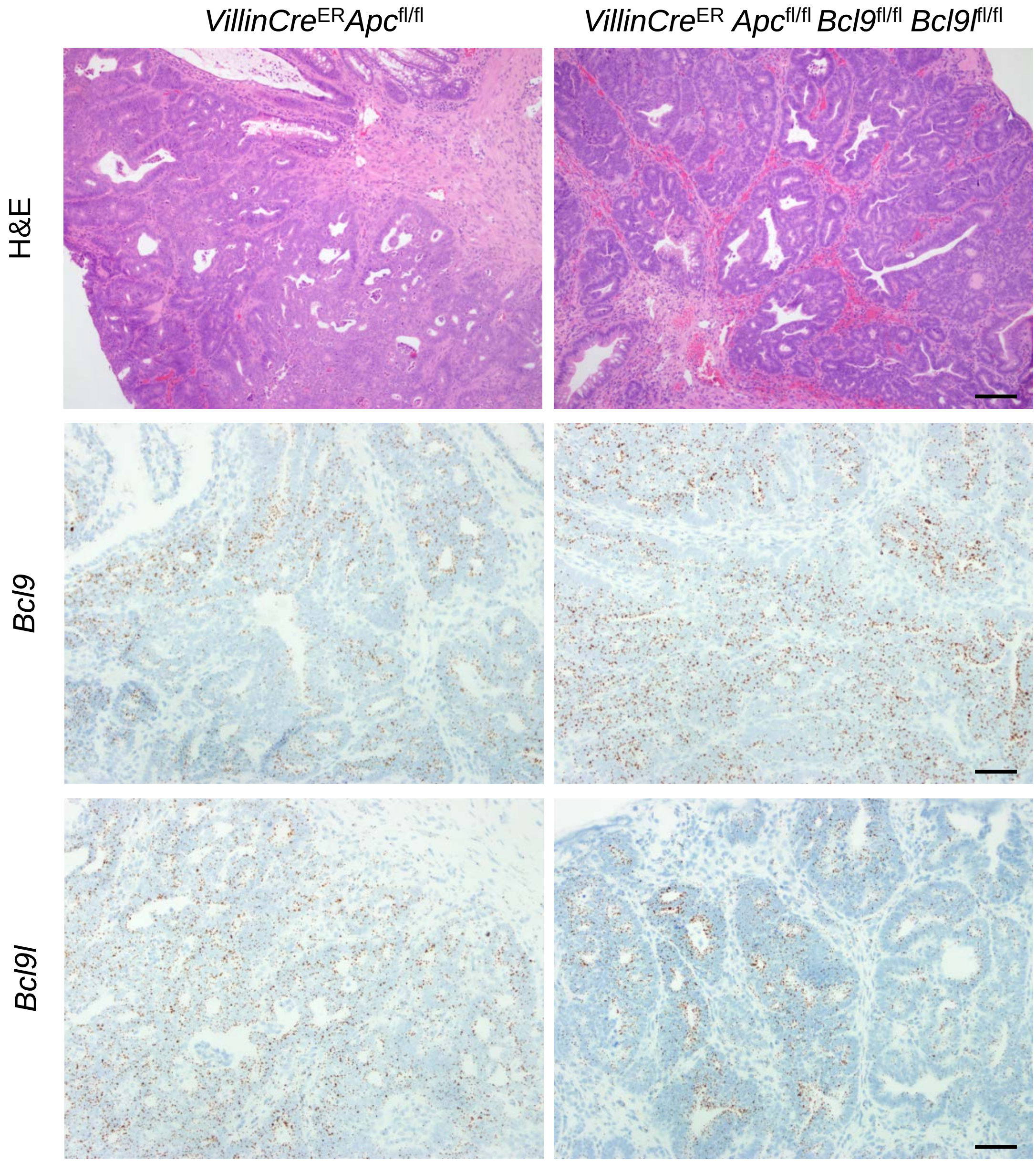

Supplementary figure 6: BCL9/9l are required for colonic tumour growth

a. Survival curve from for *VillinCre*^{ER} *Apc*$^{fl/fl}$ and *VillinCre*^{ER} *Apc*$^{fl/fl}$ *Bcl9*$^{fl/fl}$ *Bcl9l*$^{fl/fl}$ induced with a single injection of 4-hydroxytamoxifen into the colonic sub-mucosa, n=7 for *VillinCre*^{ER} *Apc*$^{fl/fl}$ (1 censors, still alive) and n=6 for *VillinCre*^{ER} *Apc*$^{fl/+}$ *Bcl9*$^{fl/fl}$ *Bcl9l*$^{fl/fl}$ (2 censors, still alive), Log-rank test P=0.0702.

b. Representative H&E, *Bcl9*- and *Bcl9l*-RNAscope staining of tumours from mice described in a. Scale bar = 100μM

Supplementary figure 7

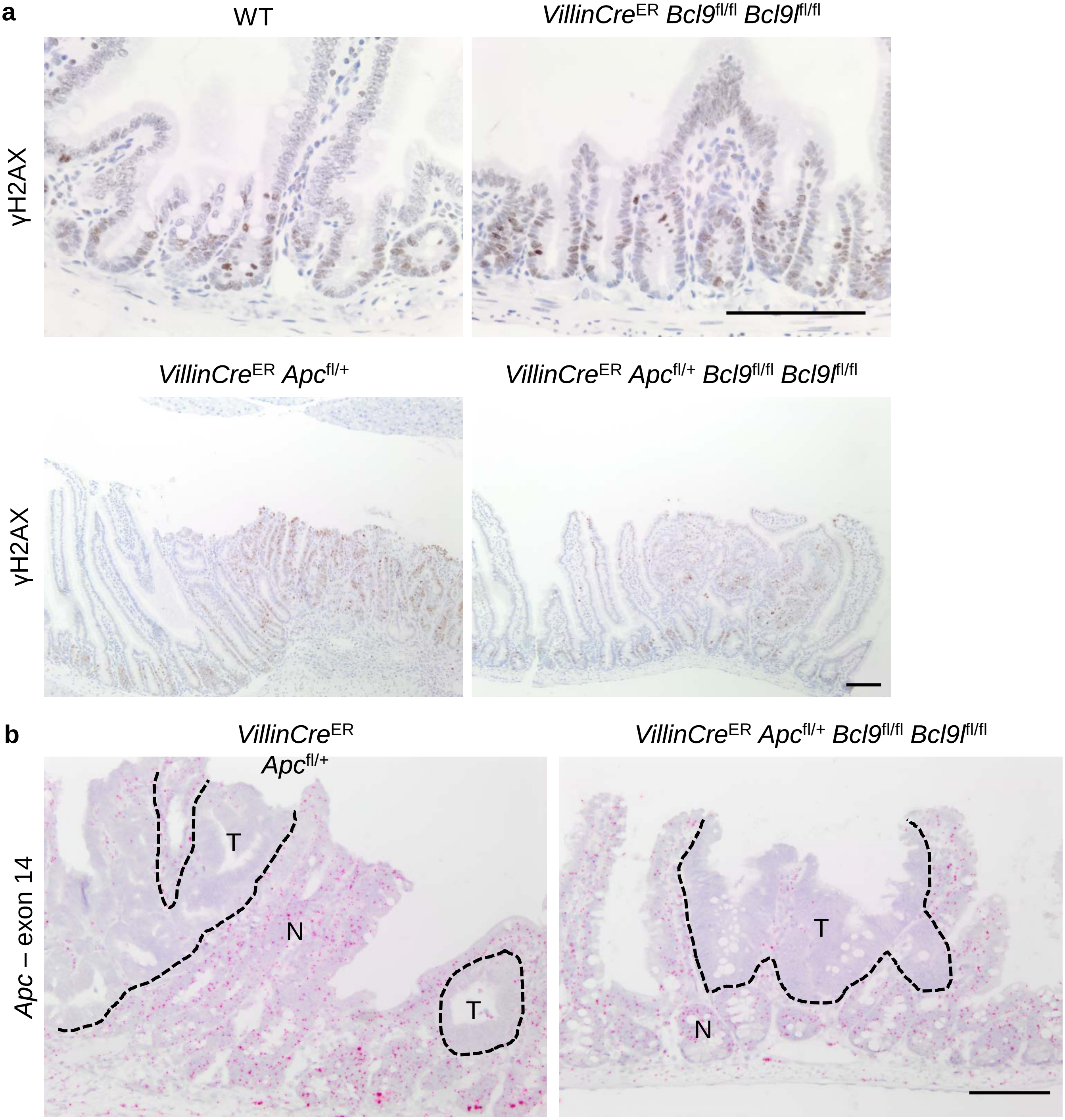

Supplementary figure 7: Deletion of *Bcl9/9l* does not increase DNA damage

a. Representative γH2Ax staining of intestinal crypts from WT and *VillinCre*^{ER} *Bcl9*$^{fl/fl}$ *Bcl9l*$^{fl/fl}$ mice sampled four days post Cre-induction (upper panel) and of small intestinal tumours from *VillinCre*^{ER} *Apc*$^{fl/+}$ and *VillinCre*^{ER} *Apc*$^{fl/+}$ *Bcl9*$^{fl/fl}$ *Bcl9l*$^{fl/fl}$ mice sampled 50 days post Cre-induction. Scale bars = 100µm

b. Representative staining of *Apc*-exon14 Basescope to detect loss of heterozygosity in intestinal tumours from Cre-induced *VillinCre*^{ER} *Apc*$^{fl/+}$ and *VillinCre*^{ER} *Apc*$^{fl/+}$ *Bcl9*$^{fl/fl}$ *Bcl9l*$^{fl/fl}$ mice sampled 50 days post-induction. Dashed line indicates border between tumour (T) and normal epithelium (N). Scale bar = 100µm.

Supplementary figure 8

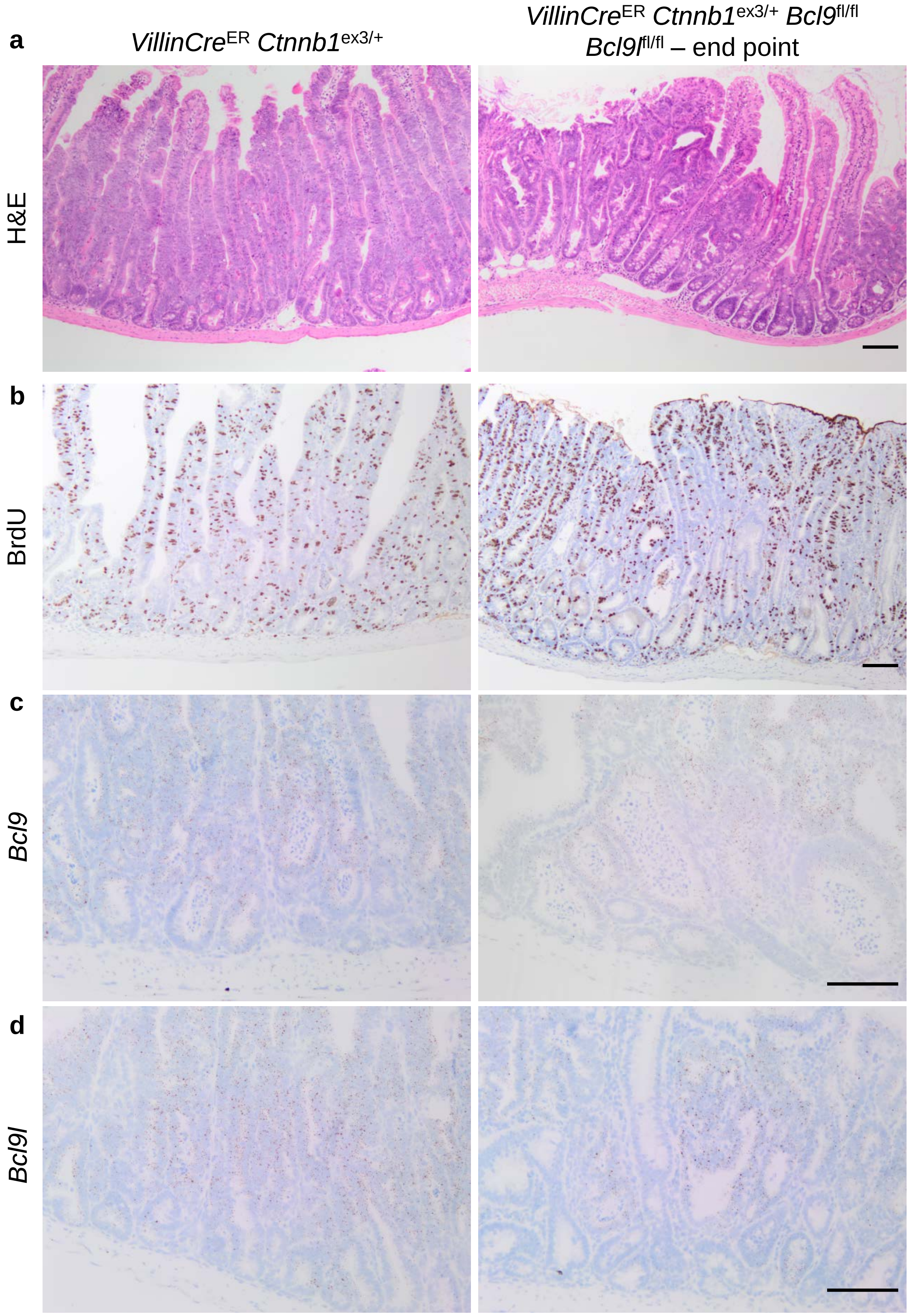

Supplementary figure 8: BCL9/9l are required for mutant β-catenin driven intestinal tumourigenesis

a. Representative H&E staining of small intestinal sections from Cre-induced *VillinCre*^{ER} *Ctnnb1*$^{ex3/+}$ and *VillinCre*^{ER} *Ctnnb1*$^{ex3/+}$ *Bcl9*$^{fl/fl}$ *Bcl9l*$^{fl/fl}$ mice sampled at clinical endpoint. Scale bars = 100µm.
b. Representative BrdU staining of small intestinal sections from mice described in A. Mice were injected with BrdU intraperitoneally 2 hours prior to being culled. Scale bars = 100µm.
c. Representative *Bcl9*-RNAscope staining of mice described in A. Scale bar = 100 µm.
d. Representative *Bcl9l*-RNAscope staining of mice described in A. Scale bar = 100 µm.

Supplementary figure 9

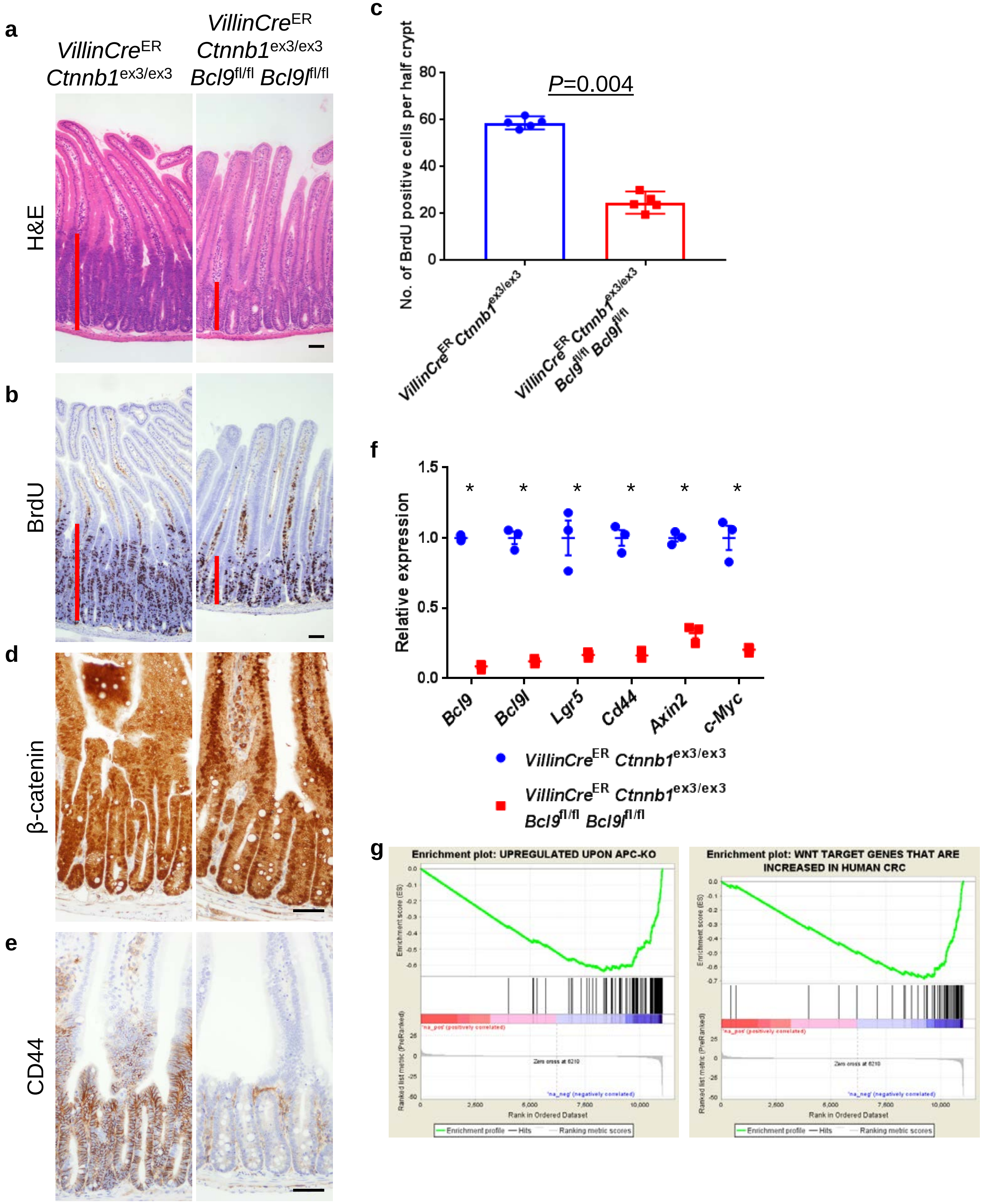

Supplementary figure 9: *Bcl9/9l* deletion suppresses acute transformation of the small intestine following mutant β-catenin expression

a. Representative H&E staining of small intestines from Cre-induced *VillinCre*^{ER} *Ctnnb1*$^{ex3/ex3}$ and *VillinCre*^{ER} *Ctnnb1*$^{ex3/ex3}$ *Bcl9*$^{fl/fl}$ *Bcl9l*$^{fl/fl}$ mice sampled four days post-induction. Red bars indicate the size of the proliferative crypt. Scale bar = 50µm.

b. Representative BrdU staining of mice described in a. Mice were injected intraperitoneally with BrdU 2 hours prior to being culled. Red bars indicated the size of the proliferative crypt. Scale bar = 50µm

c. Quantification of proliferation (BrdU positive cells) in the small intestines of mice described in a. The number of BrdU-positive cells per half crypt was quantified, 25 crypts per mouse scored, n=5 for each group, one-way Mann-Whitney *U* test *P*=0.004. Data displayed as mean ± SEM.

d. Representative β-catenin staining of mice described in a.

e. Representative CD44 staining of mice described in a.

f. qPCR for Wnt target genes and intestinal stem cell markers from intestinal tissue of *VillinCre*^{ER} *Ctnnb1*$^{ex3/ex3}$ and *VillinCre*^{ER} *Ctnnb1*$^{ex3/ex3}$ *Bcl9*$^{fl/fl}$ *Bcl9l*$^{fl/fl}$ mice, n=3 per group, one-way Mann-Whitney *U* test, * (*P*=0.04). Data displayed as relative to the mean of *VillinCre*^{ER} *Ctnnb1*$^{ex3/ex3}$ mice. Data displayed as mean ± SEM.

g. Gene Set Enrichment Analysis of RNAseq data from small intestinal tissue from *VillinCre*^{ER} *Ctnnb1*$^{ex3/ex3}$ and *VillinCre*^{ER} *Ctnnb1*$^{ex3/ex3}$ *Bcl9*$^{fl/fl}$ *Bcl9l*$^{fl/fl}$ mice, n=3 mice per group.

Supplementary figure 10

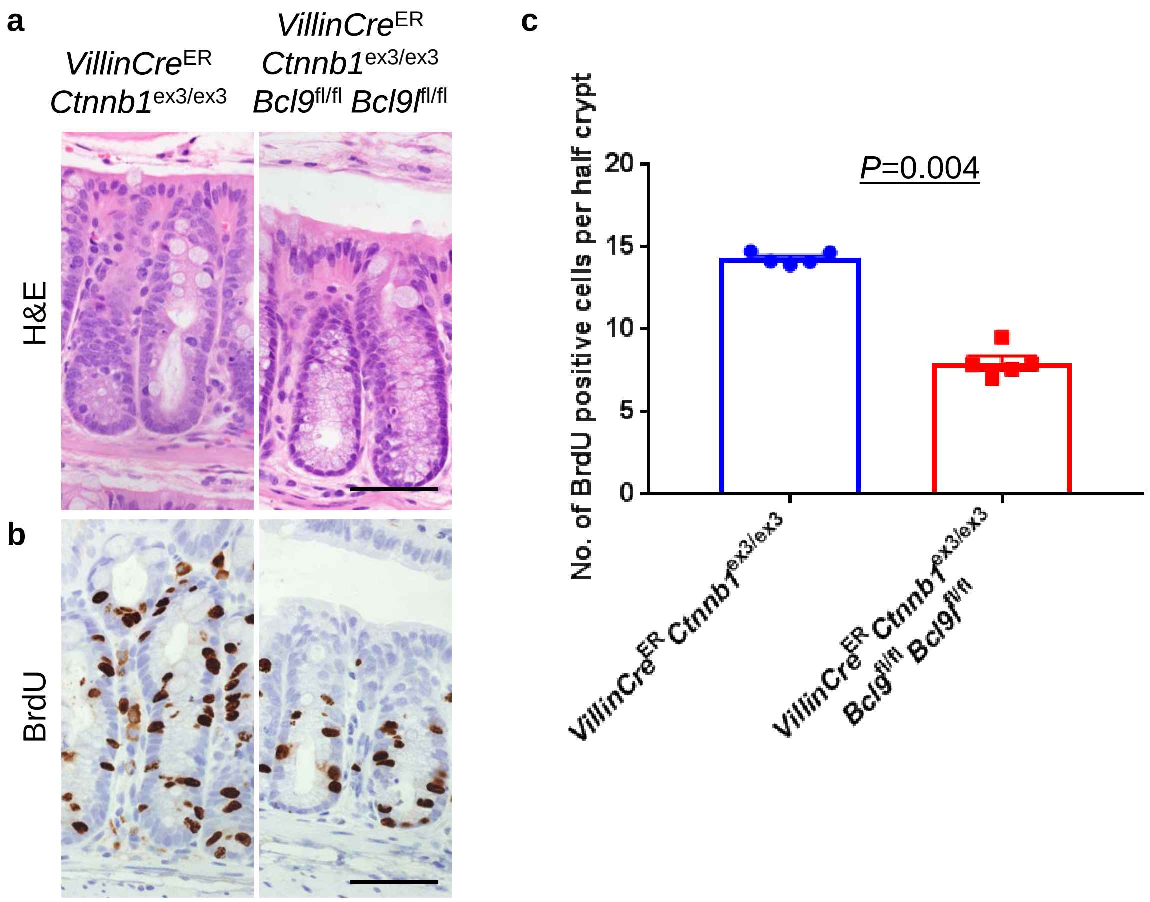

Supplementary figure 10: *Bcl9/9l* deletion suppresses acute transformation of the colon following mutant β-catenin expression

a. Representative H&E staining of colonic sections from *VillinCre*^{ER} *Ctnnb1*$^{ex3/ex3}$ and *VillinCre*^{ER} *Ctnnb1*$^{ex3/ex3}$ *Bcl9*$^{fl/fl}$ *Bcl9l*$^{fl/fl}$ mice sampled four days post Cre-induction. Scale bar = 50µm.

b. Representative BrdU staining of mice described in a. Mice were injected with intraperitoneally with BrdU 2 hours prior to being culled. Scale bar = 50µm

c. Quantification of proliferation (BrdU positive cells) in the small intestines of mice described in a. The number of BrdU-positive cells per half crypt quantified, 25 crypts per mouse scored, n=5 for each group, one-way Mann-Whitney *U* test *P*=0.004. Data displayed as mean ± SEM.

Supplementary figure 11

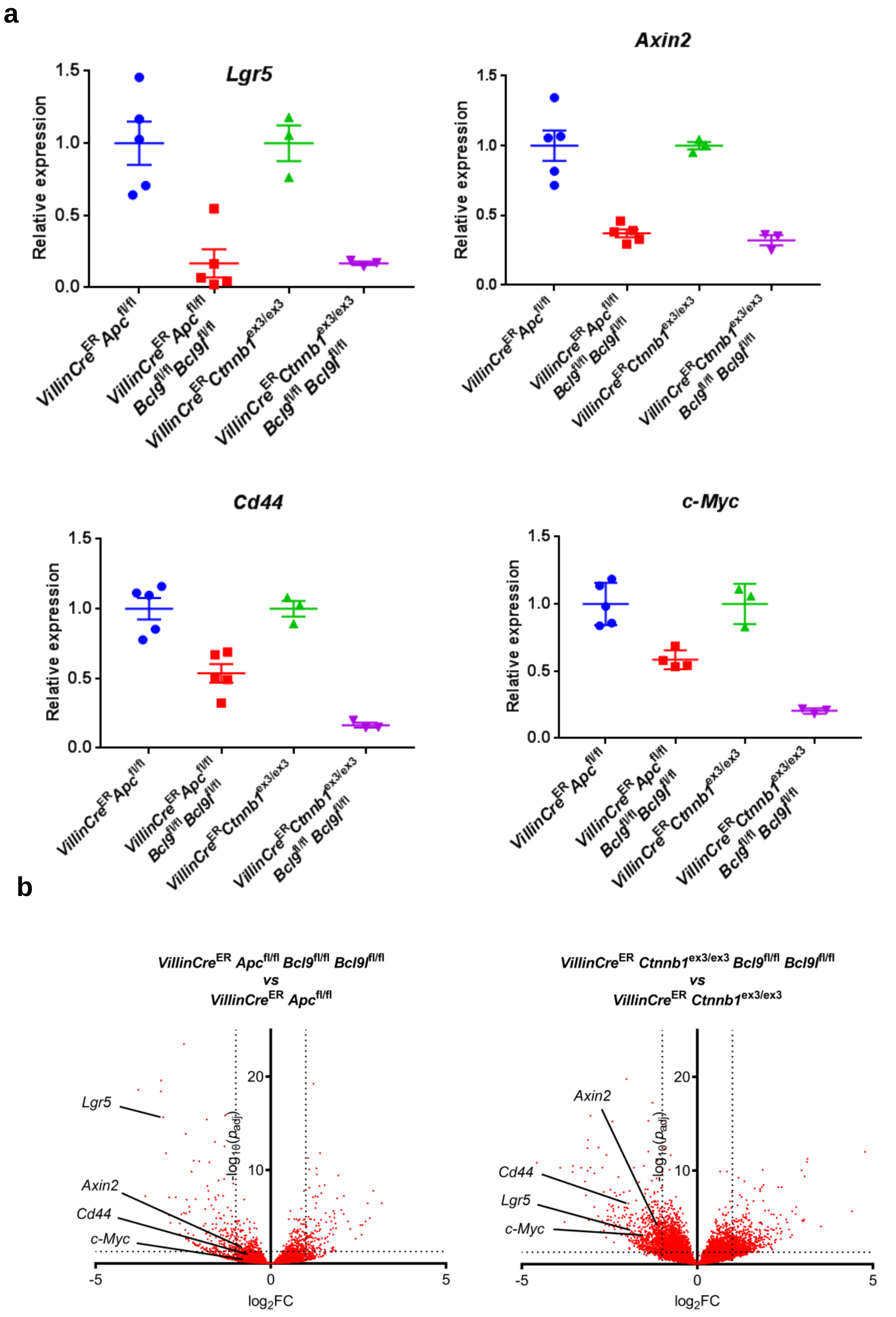

Supplementary figure 11: *Bcl9/9l* deletion preferentially reduces the expression a subset of Wnt target genes following β-catenin mutation compared with APC loss

a. qPCR for *Lgr5*, *Axin2*, *Cd44* and *c-Myc* from intestinal tissue of Cre-induced *VillinCre*^{ER} *Apc*$^{fl/fl}$, *VillinCre*^{ER} *Apc*$^{fl/fl}$ *Bcl9*$^{fl/fl}$ *Bcl9l*$^{fl/fl}$, *VillinCre*^{ER} *Ctnnb1*$^{ex3/ex3}$ and *VillinCre*^{ER} *Ctnnb1*$^{ex3/ex3}$ *Bcl9*$^{fl/fl}$ *Bcl9l*$^{fl/fl}$ mice sampled four days post Cre-induction, n=3-5 per group. Data displayed as relative expression normalised to either Cre-induced *VillinCre*^{ER} *Apc*$^{fl/fl}$ or *VillinCre*^{ER} *Ctnnb1*$^{ex3/ex3}$. Data displayed as mean ± SEM. Data same as Fig. 4h and Supplementary Figure 9f.

b. Volcano plots for RNAseq data from Cre-induced *VillinCre*^{ER} *Apc*$^{fl/fl}$ vs *VillinCre*^{ER} *Apc*$^{fl/fl}$ *Bcl9*$^{fl/fl}$ *Bcl9l*$^{fl/fl}$ and *VillinCre*^{ER} *Ctnnb1*$^{ex3/ex3}$ and *VillinCre*^{ER} *Ctnnb1*$^{ex3/ex3}$ *Bcl9*$^{fl/fl}$ *Bcl9l*$^{fl/fl}$ mice. Dashed lines intersecting the x and y-axes represent a fold change of 2 or *P* value of 0.05 respectively. The positions of *Lgr5*, *Axin2*, *Cd44* and *c-Myc* are indicated on each plot. 4 genes lie outside of the displayed graph for the *VillinCre*^{ER} *Ctnnb1*$^{ex3/ex3}$ and *VillinCre*^{ER} *Ctnnb1*$^{ex3/ex3}$ *Bcl9*$^{fl/fl}$ *Bcl9l*$^{fl/fl}$ volcano plot: *Hsd3b3* [$\log_2$FC = 5.74 and $\log_{10}(P_{adj})$ = 10.8], *Gm8960* [$\log_2$FC = 5.4 and $\log_{10}(P_{adj})$ = 43.3], *Rps3a1* [$\log_2$FC = 5.3 and $\log_{10}(P_{adj})$ = 80.5] and *Jaml* [$\log_2$FC = 3.46 and $\log_{10}(P_{adj})$ = 107.3].

Supplementary figure 12

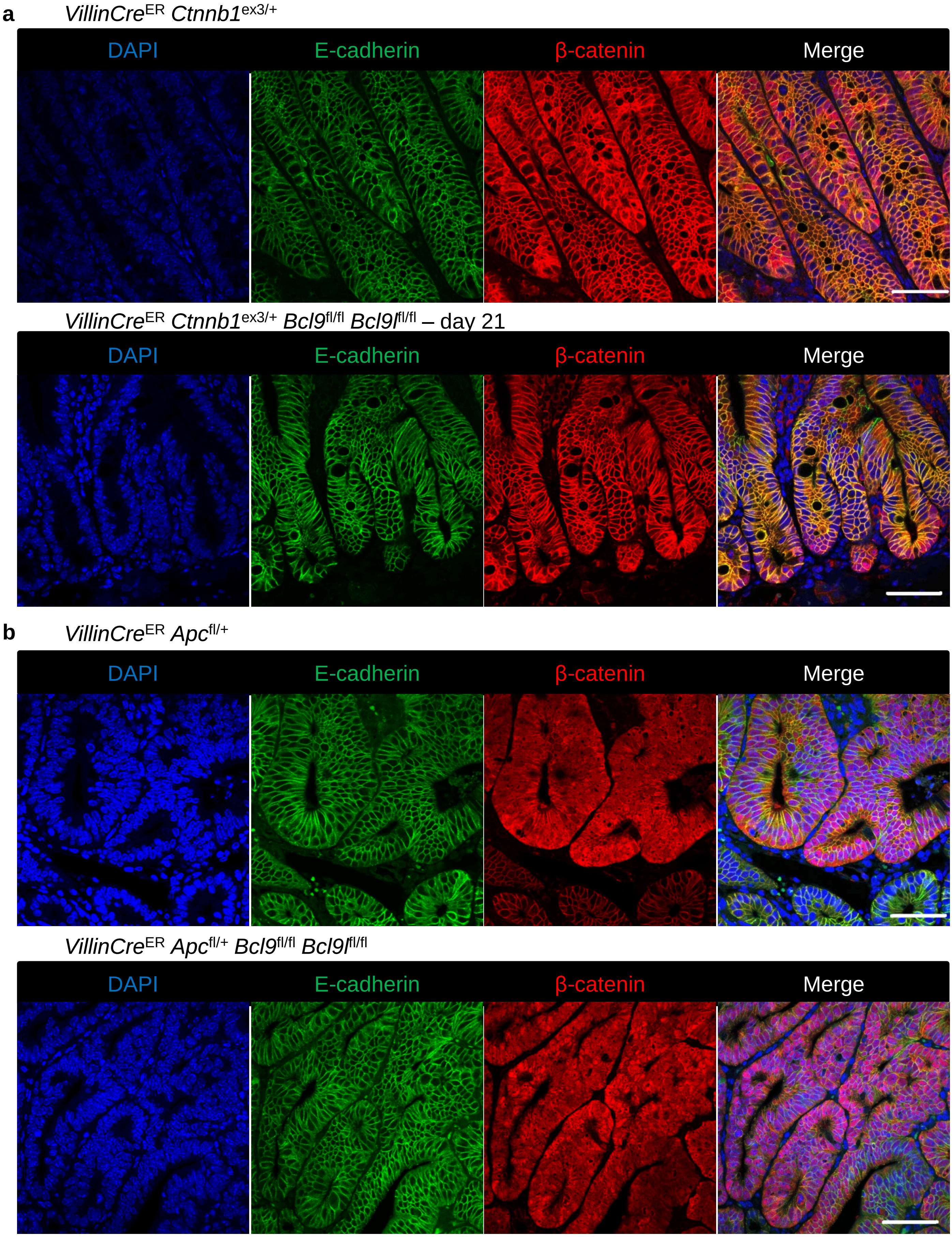

Supplementary Figure 12: *Bcl9/9l* deletion increases membranous β-catenin in β-catenin mutant crypts

a. Representative immunofluorescent staining for DAPI - nuclei (blue), E-cadherin (green) and β-catenin (red) of intestinal crypts from Cre-induced *VillinCre*^{ER} *Ctnnb1*$^{ex3/+}$ (sampled at end-point) and *VillinCre*^{ER} *Ctnnb1*$^{ex3/+}$ *Bcl9*$^{fl/fl}$ *Bcl9l*$^{fl/fl}$ mice (sampled at day 21). Scale bar = 50μm.

b. Representative immunofluorescent staining for DAPI – nuclei (blue), E-cadherin (green) and β-catenin (red) of intestinal tumours from *VillinCre*^{ER} *Apc*$^{fl/+}$ and *VillinCre*^{ER} *Apc*$^{fl/+}$ *Bcl9*$^{fl/fl}$ *Bcl9l*$^{fl/fl}$ mice sampled at end-point. Scale bar = 50μm.

Supplementary figure 13

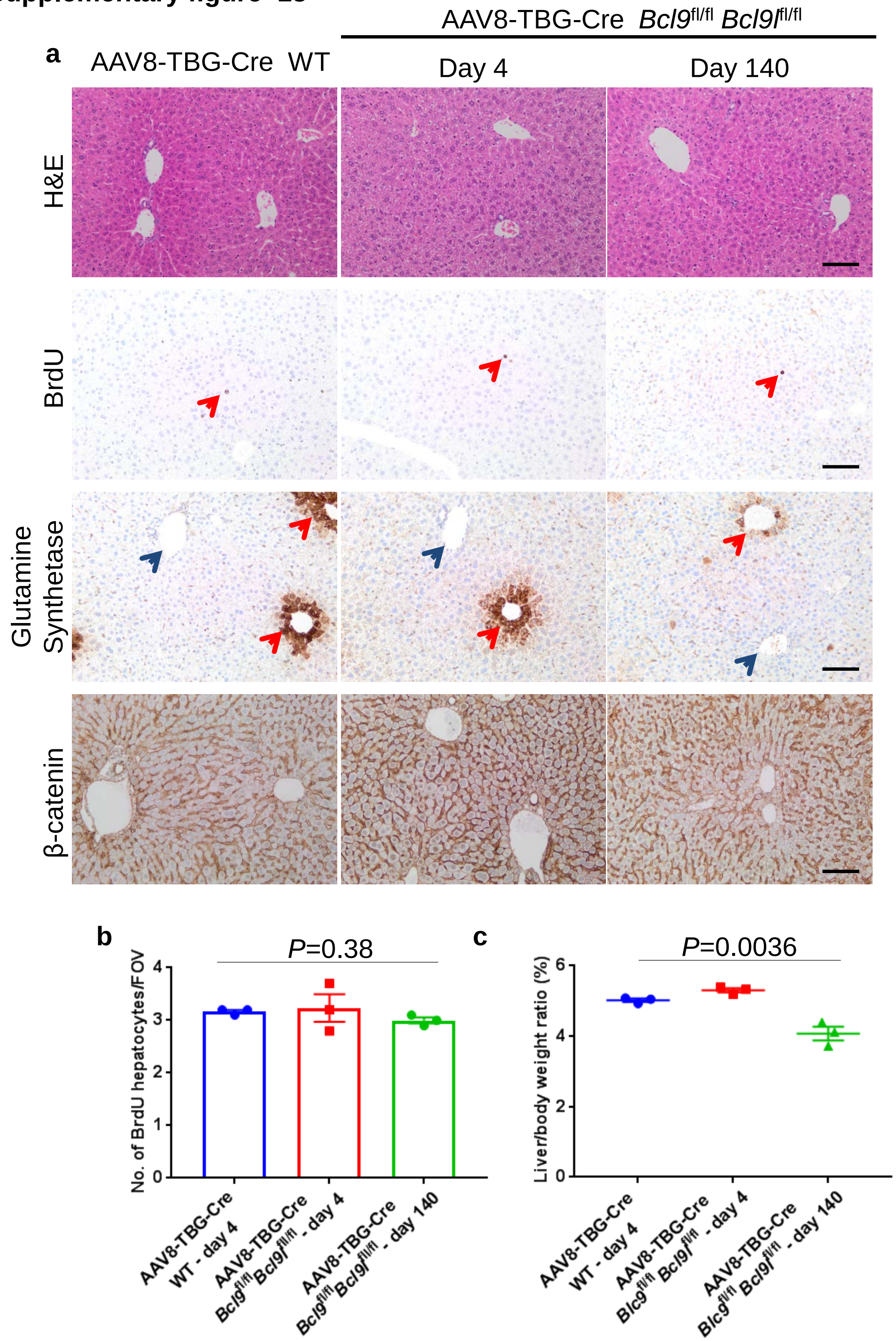

Supplementary figure 13: BCL9/9l are dispensable in the liver
a. Representative H&E, BrdU (red arrows highlight positive hepatocytes), Glutamine Synthetase (GS; red and blue arrows highlight central vein and portal tract areas respectively) and β-catenin of liver sections from AAV-TBG-Cre induced WT and *Bcl9*$^{fl/fl}$ *Bcl9l*$^{fl/fl}$ mice sampled four days post-induction or aged for 140 days. Scale bar = 50µm.
b. Quantification of hepatocyte proliferation in AAV8-TBG-Cre induced WT and *Bcl9*$^{fl/fl}$ *Bcl9l*$^{fl/fl}$ mice livers sampled four days post-induction or aged for 140 days. Mice were injected with BrdU intraperitoneally 2 hours prior to culling. Number of BrdU positive hepatocytes scored per 10x field of view, 10 fields scored per mouse n=3 per group, Kruskal-Wallis test, *P*=0.38. Data displayed as mean $\pm$ SEM.
c. Liver-to-body weight ratio (%) of AAV8-TBG-Cre induced WT and *Bcl9*$^{fl/fl}$ *Bcl9l*$^{fl/fl}$ mice sampled four days post-induction or aged for 140 days, n=3 per group, Kruskal-Wallis test, *P*=0.0036. Data displayed as mean $\pm$ SEM.

Supplementary figure 14

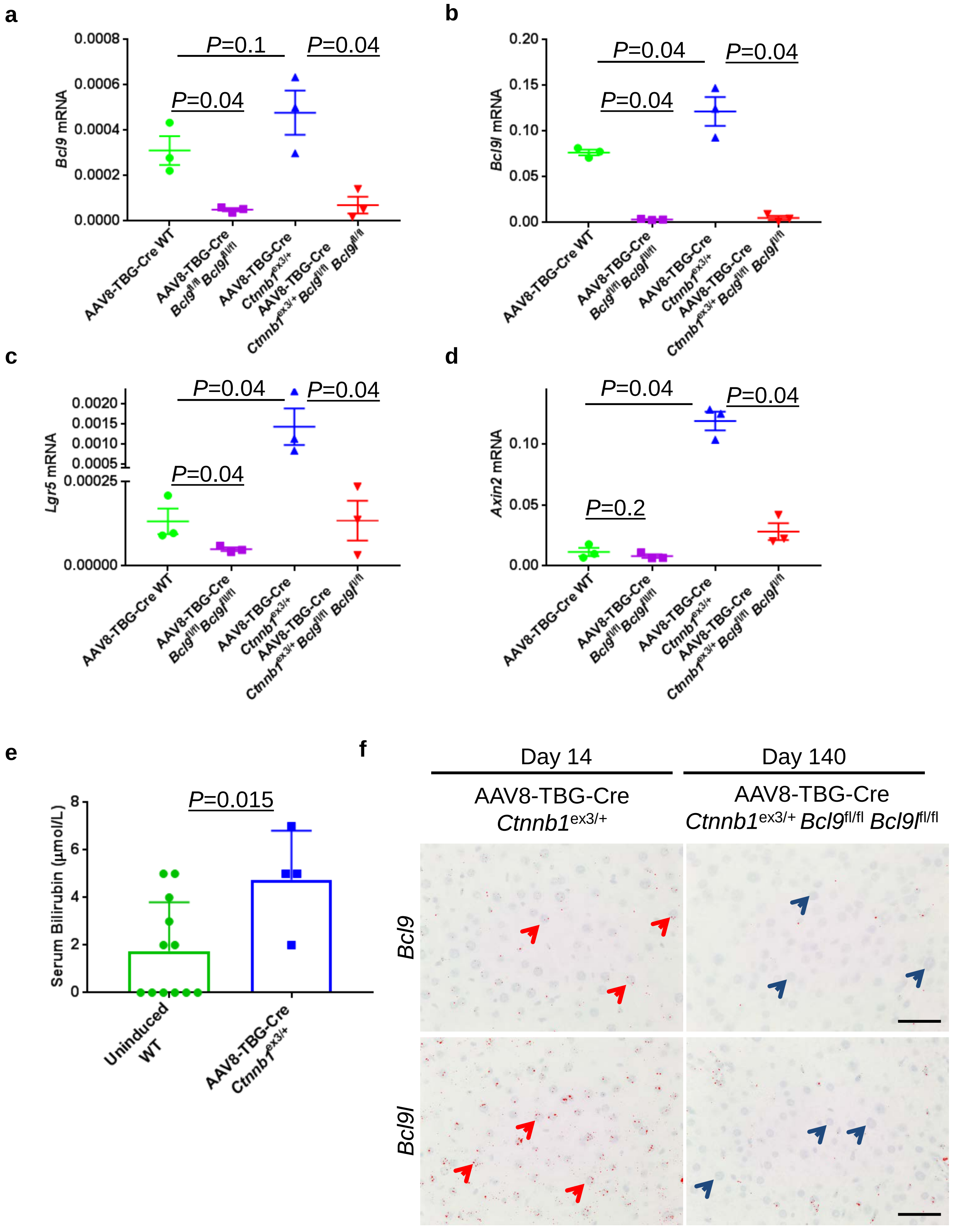

Supplementary figure 14: BCL9/9l are required for β-catenin driven hepatocyte transformation

a-d. qRT-PCR for a. *Bcl9* b. *Bcl9l* c. *Lgr5* and d. *Axin2* on liver pieces from AAV8-TBG-Cre WT, AAV8-TBG-Cre *Bcl9*$^{fl/fl}$ *Bcl9l*$^{fl/fl}$, AAV8-TBG-Cre *Ctnnb1*$^{ex3/+}$ and AAV8-TBG-Cre *Ctnnb1*$^{ex3/+}$ *Bcl9*$^{fl/fl}$ *Bcl9l*$^{fl/fl}$ sampled 4-days post induction, n=3 per group, one-way Mann-Whitney *U* test. Data displayed as mean $\pm$ SEM.

e. Serum bilirubin levels isolated from uninduced WT and AAV8-TBG-Cre *Ctnnb1*$^{ex3/+}$ mice sampled at end point, n= 12 and 4, one-way Mann-Whitney *U* test *P*=0.015. Data displayed as mean $\pm$ SEM.

f. *Bcl9*- (upper panel) and *Bcl9l*-RNAscope (lower panel) staining of liver sections from AAV8-TBG-Cre *Ctnnb1*$^{ex3/+}$ mice sampled at day 14 and AAV8-TBG-Cre *Ctnnb1*$^{ex3/+}$ *Bcl9*$^{fl/fl}$ *Bcl9l*$^{fl/fl}$ mice sampled at day 140. Red arrows indicate hepatocytes positive for *Bcl9* and *Bcl9l*, whilst blue arrows indicate hepatocytes that are negative for *Bcl9* and *Bcl9l* expression. Images are false colour images to enhance visualisation of RNAscope staining Scale bar = 50μm

Supplementary table s

Supplementary table 1 – GSEA from *VillinCre*^{ER} *Bcl9*$^{fl/fl}$ *Bcl9l*$^{fl/fl}$ vs WT RNAseq

**Gene Set**	**NES**	**FDR**
LGR5 HIGH VS LOW STUDY A	-3.53	0
LGR5 CROSS-PLATFORM	-2.82	0
PROGENITOR CLUSTER	-2.59	0

In relation to Figure 1e:
Gene Set Enrichment Analysis of RNAseq data obtained from small intestinal tissue from WT and *VillinCre*^{ER} *Bcl9*$^{fl/fl}$ *Bcl9l*$^{fl/fl}$ mice. Table showing negatively enriched gene sets of RNAseq data from WT and *VillinCre*^{ER} *Bcl9*$^{fl/fl}$ *Bcl9l*$^{fl/fl}$ mice, n=3 per group.

Supplementary table 2 – GSEA from *VillinCre*^{ER} *Apc*$^{fl/fl}$ *Bcl9*$^{fl/fl}$ *Bcl9l*$^{fl/fl}$ vs *VillinCre*^{ER} *Apc*$^{fl/fl}$ RNAseq

Gene set	NES	FDR
UPREGULATED UPON APC-KO	-5.84	0
WNT TARGET GENES THAT ARE INCREASED IN HUMAN CRC	-4.22	0
DIRECT AND FUNCTIONAL BETA-CATENIN TARGETS IN SW480	-1.62	0.04

In relation to Figure 4d:
Table showing negatively enriched gene sets of RNAseq data from Cre-induced *VillinCre*^{ER} *Apc*$^{fl/fl}$ *Bcl9*$^{fl/fl}$ *Bcl9l*$^{fl/fl}$ vs *VillinCre*^{ER} *Apc*$^{fl/fl}$ mice.

Supplementary table 3 – GSEA from *VillinCre*^{ER} *Ctnnb1*$^{ex3/ex3}$ *Bcl9*$^{fl/fl}$ *Bcl9l*$^{fl/fl}$ vs *VillinCre*^{ER} *Ctnnb1*$^{ex3/ex3}$ RNAseq

Gene set	NES	FDR
UPREGULATED UPON APC-KO	-7.53	0
WNT TARGET GENES THAT ARE INCREASED IN HUMAN CRC	-6.69	0

In relation to Supplementary figure 9g:
Table shows negatively enriched gene sets in *VillinCre*^{ER} *Ctnnb1*$^{ex3/ex3}$ *Bcl9*$^{fl/fl}$ *Bcl9l*$^{fl/fl}$ vs *VillinCre*^{ER} *Ctnnb1*$^{ex3/ex3}$ small intestines.

Supplementary table 4 – qPCR primers

Ctnnb1	Fwd	ATC TTA AGC CCT CGC TCG GT
	Rev	CTT CAG GTA CCC TCAG GCC C
Cdh1	Fwd	ACT GTG AAG GGA CGG TCA AC
	Rev	GGA GCA GCA GGA TCA GAA TC
Axin2	Fwd	GCG ACG CAC TGA CCG ACG AT
	Rev	GCA GGC GGT GGG TTC TCG GA
Cd44	Fwd	CAC ATA TTG CTT CAA TGC CTC AG
	Rev	CCA TCA CGG TTG ACA ATA GTT ATG
Lgr5	Fwd	GAC AAT GCT CTC ACA GAC
	Rev	GGA GTG GAT TCT ATT ATT ATG G
Gapdh	Fwd	GAA GGC CGG GGC CCA CTT GA
	Rev	CTG GGT GGC AGT GAT GGC ATG G
Bcl9	Fwd	AGT GCT CTC TCC AGG ATA TGA TG
	Rev	GGG CAA AGA TGT TGA AAT GTT G
Bcl9l	Fwd	AGC AGC ACC TAA TGG GCA AAG
	Rev	GGA TAA GTC GAA CTC AGG AAT GC
cMyc	Fwd	CCC AAA TCC TGT ACC TCG TC
	Rev	TTG CCT CTT CTC CAC AGA CA
Lrig1	Fwd	Primerdesign
	Rev	Primerdesign
