# Supplementary material for: Loss of BCL9/9l suppresses Wnt driven tumourigenesis in models that recapitulate human cancer
Source: Nat Commun. 2019 Feb 13;10:723. doi: 10.1038/s41467-019-08586-3 (PMC6374445; doi:10.1038/s41467-019-08586-3)
Supplement: Supplementary file 3 — Reporting Summary [file 41467_2019_8586_MOESM3_ESM.pdf]

nature research

Corresponding author(s): DBPR - NCOMMS-18-18031A

Last updated by author(s): Dec 10, 2018

Reporting Summary

Nature Research wishes to improve the reproducibility of the work that we publish. This form provides structure for consistency and transparency in reporting. For further information on Nature Research policies, see Authors & Referees and the Editorial Policy Checklist.

Statistics

For all statistical analyses, confirm that the following items are present in the figure legend, table legend, main text, or Methods section.

n/a	Confirmed	
☐	☒	The exact sample size (*n*) for each experimental group/condition, given as a discrete number and unit of measurement
☐	☒	A statement on whether measurements were taken from distinct samples or whether the same sample was measured repeatedly
☐	☒	The statistical test(s) used AND whether they are one- or two-sided<br>*Only common tests should be described solely by name; describe more complex techniques in the Methods section.*
☒	☐	A description of all covariates tested
☒	☐	A description of any assumptions or corrections, such as tests of normality and adjustment for multiple comparisons
☐	☒	A full description of the statistical parameters including central tendency (e.g. means) or other basic estimates (e.g. regression coefficient) AND variation (e.g. standard deviation) or associated estimates of uncertainty (e.g. confidence intervals)
☐	☒	For null hypothesis testing, the test statistic (e.g. *F*, *t*, *r*) with confidence intervals, effect sizes, degrees of freedom and *P* value noted<br>*Give P values as exact values whenever suitable.*
☒	☐	For Bayesian analysis, information on the choice of priors and Markov chain Monte Carlo settings
☒	☐	For hierarchical and complex designs, identification of the appropriate level for tests and full reporting of outcomes
☒	☐	Estimates of effect sizes (e.g. Cohen's *d*, Pearson's *r*), indicating how they were calculated

Our web collection on statistics for biologists contains articles on many of the points above.

Software and code

Policy information about availability of computer code

Data collection

No software was used

Data analysis

ImageJ 1.48v; Java 1.6.0_20 was used to analyze the Proximity Ligation Assay
All statistics were performed using GraphPad Prism V6 Software (La Jolla, CA, USA)
RNAseq:
Quality checks on the raw RNASeq data files were done using fastqc (v0.10.1) [http://www.bioinformatics.bbsrc.ac.uk/projects/fastqc] and fastq_screen (v0.4.2) [http://www.bioinformatics.babraham.ac.uk/projects/fastq_screen/]. RNASeq reads were aligned to the GRCm38 (Church et al., 2011) version of the mouse genome using tophat(v2.0.13) (D. Kim et al., 2013) with Bowtie (v2.2.4.0) (Langmead & Salzberg, 2012). Relative expression levels were determined and statistically analysed using a combination of HTSeq-count (version 0.6.1p1) [http://www-huber.embl.de/users/anders/HTSeq/doc/overview.html], the R statistical analysis environment (version 3.2.2), utilizing packages from the Bioconductor data
analysis suite. Data normalisation and differential gene expression analysis are based on a generalized linear model using the DESeq2 package (Love et al., 2014).

Church, D. M., Schneider, V. A., Graves, T., Auger, K., Cunningham, F., Chen, H., … Hubbard, T. (2011). Modernizing Reference Genome Assemblies, 9(7), 1–5. https://doi.org/10.1371/journal.pbio.1001091
Kim, D., Pertea, G., Trapnell, C., Pimentel, H., Kelley, R., & Salzberg, S. L. (2013). TopHat2?: accurate alignment of transcriptomes in the presence of insertions , deletions and gene fusions, 1–13.
Langmead, B., & Salzberg, S. L. (2012). Fast gapped-read alignment with Bowtie 2, 9(4), 357–360. https://doi.org/10.1038/nmeth.1923
Love, M. I., Huber, W., & Anders, S. (2014). Moderated estimation of fold change and dispersion for RNA-seq data with DESeq2, 1–21. https://doi.org/10.1186/s13059-014-0550-8

For manuscripts utilizing custom algorithms or software that are central to the research but not yet described in published literature, software must be made available to editors/reviewers. We strongly encourage code deposition in a community repository (e.g. GitHub). See the Nature Research guidelines for submitting code & software for further information.

Data

Policy information about availability of data

All manuscripts must include a data availability statement. This statement should provide the following information, where applicable:

- Accession codes, unique identifiers, or web links for publicly available datasets
- A list of figures that have associated raw data
- A description of any restrictions on data availability

The authors declare that all relevant data supporting the findings of this study are available within the article and its Supplementary Information files. Accession codes will be available before publication.

Field-specific reporting

Please select the one below that is the best fit for your research. If you are not sure, read the appropriate sections before making your selection.

☒ Life sciences ☐ Behavioural & social sciences ☐ Ecological, evolutionary & environmental sciences

For a reference copy of the document with all sections, see nature.com/documents/nr-reporting-summary-flat.pdf

Life sciences study design

All studies must disclose on these points even when the disclosure is negative.

Sample size

In accordance with the 3Rs, the smallest sample size was chosen that could give a significant difference. Given the robust phenotypes of the Apcfl/fl model, and our prediction that BCL9 and BCL9l were essential, the minimum sample size assuming no overlap in control versus experimental is three animals. Given that the Bcatex3/ex3 model shows similar kinetics and phenotype to Apcfl/fl - we also felt that the minimum number of experimental animals is three, but with a maximum of five.
For Apcfl/+ survival curve, we performed a power analysis:
We assumed that control and experimental groups have a similar standard deviation, and that to observe a difference in median survival of 20% you would need 13 animals per group:
t tests - Means: Wilcoxon-Mann-Whitney test (two groups)
Options: A.R.E. method
Analysis: A priori: Compute required sample size
Input: Tail(s) = One
Parent distribution = Normal
Effect size d = 1.0333333
α err prob = 0.05
Power (1-β err prob) = 0.8
Allocation ratio N2/N1 = 1
Output: Noncentrality parameter δ = 2.5744401
Critical t = 1.7144127
Df = 22.8281711
Sample size group 1 = 13
Sample size group 2 = 13
Total sample size = 26
Actual power = 0.8029198

We have observed a difference in survival of just under 20%, so a control group of 14 mice is sufficient.
A post-hoc analysis confirms that we have achieved a power of 86%, exceeding the defined 80%:
t tests - Means: Wilcoxon-Mann-Whitney test (two groups)
Options: A.R.E. method
Analysis: Post hoc: Compute achieved power
Input: Tail(s) = One
Parent distribution = Normal
Effect size d = 1.1200000
α err prob = 0.05
Sample size group 1 = 13
Sample size group 2 = 14
Output: Noncentrality parameter δ = 2.8415645
Critical t = 1.7115083
Df = 23.7831008
Power (1-β err prob) = 0.8674500

In our study, we observed a difference in survival of approximately 30%, using 16 and 19 animals per cohort – however it is important to note that 3 animals had to be censored (as outlined below) in the VillinCreER Apcfl/+ cohort – hence the slightly larger sample size in this study.
For B-catenin driven intestinal and liver models, we hypothesized a much larger difference in survival following deletion of BCL9/9l, but also from previous studies, it was known that the control arms have a very small standard deviation in survival and therefore we needed feweer mice in each cohort. Subsequently, we aimed for 5-8 mice per cohort in order to see a difference in survival.

Data exclusions	No data was excluded, however in figure 5 a - three mice were censored due to: 1 mouse had lymphoma, 1 with elongated teeth and another displayed rapid weight loss without significant tumour burden. We determined that these mice had not succumbed to a phenotype associated with intestinal tumour burden
Replication	For acute mouse models - VillinCreER Apcfl/fl and VillinCreER Ctnnb1ex3/ex3 - data was combined from independent mice, induced on different days and analyzed together - showing the same phenotype from each batch following deletion of BLC9 and BLC9l.
Randomization	No randomization was used
Blinding	The researcher was blinded to samples/mice during husbandry and analysis, such as quantification/analysis of IHC staining.

Reporting for specific materials, systems and methods

We require information from authors about some types of materials, experimental systems and methods used in many studies. Here, indicate whether each material, system or method listed is relevant to your study. If you are not sure if a list item applies to your research, read the appropriate section before selecting a response.

Materials & experimental systems

n/a	Involved in the study
☐	☒ Antibodies
☒	☐ Eukaryotic cell lines
☒	☐ Palaeontology
☐	☒ Animals and other organisms
☒	☐ Human research participants
☒	☐ Clinical data

Methods

n/a	Involved in the study
☒	☐ ChIP-seq
☒	☐ Flow cytometry
☒	☐ MRI-based neuroimaging

Antibodies

Antibodies used	Primary antibodies used for immunohistochemistry were as follows: BrdU (1:200, BD Biosciences #347580), SOX9 (1:500, Chemicon #AB5535), β-catenin (1:50, BD Biosciences #610154), BCL9 (1:500, Abnova #H00000607-MO1), Glutamine Synthetase (1:200 BD Biosciences #610518), γ-H2AX (1:50, Cell Signalling Technologies #9718) and CD44 (1:50 BD Biosciences #550538). For immunofluorescence (1:200, BD Biosciences #610154) and E-cadherin (1:200, Cell Signalling technologies #3195). For Proximity Ligation assay - E-cadherin (1:200, R&D Systems AF748) and β-catenin (1:2000, #610154, BD Biosciences).
Validation	All antibodies have been previously validated by the manufacturer.<br>Whilst within our study, our genetic models served as positive/negative controls for some of the stains. Such as deletion of BCL9. Whilst the reduction in Wnt signalling associated with deletion of BCL9/9l was reflected by reduced expression of known Wnt target genes CD44 and SOX9 in the intestine and Glutamine Synthetase in the liver. This was further supported by qRT-PCR and RNAseq data

Animals and other organisms

Policy information about studies involving animals; ARRIVE guidelines recommended for reporting animal research

Laboratory animals	Male and female C57BL/6J >20g mice were induced with tamoxifen from 6 to 12 weeks of age
Wild animals	N/A
Field-collected samples	N/A
Ethics oversight	All experiments were performed according to UK Home Office regulations (licence 70/8646), and reviewed by local ethical review committee at the University of Glasgow.

Note that full information on the approval of the study protocol must also be provided in the manuscript.
